# In Vivo Biofilm Formation of Pathogenic *Leptospira* spp. in the Vitreous Humor of Horses with Recurrent Uveitis

**DOI:** 10.3390/microorganisms9091915

**Published:** 2021-09-09

**Authors:** Kerstin Ackermann, Rebecca Kenngott, Monica Settles, Hartmut Gerhards, Johann Maierl, Bettina Wollanke

**Affiliations:** 1 Equine Clinic, Clinical Department, Ludwig-Maximilians-University, 80539 Munich, Germany; 2 Institute for Anatomy, Histology and Embryology, Department of Veterinary Science, Ludwig-Maximilians-University, 80539 Munich, Germany; m.settles@anat.vetmed.uni-muenchen.de (M.S.); j.maierl@anat.vetmed.uni-muenchen.de (J.M.)

**Keywords:** in vivo biofilm formation, biofilm infection, *Leptospira* spp., immune tolerance, antibiotic tolerance, equine recurrent uveitis (ERU), vitreous humor, immunohistochemistry, Warthin-Starry silver stain

## Abstract

Equine recurrent uveitis (ERU) causes painful inflammatory attacks and oftentimes blindness in the affected eyes. The disease is considered a late sequela of systemic leptospirosis. The most effective therapy is the surgical removal of the vitreous (vitrectomy), which is not only therapeutic, but provides vitreous material that can be assessed diagnostically. For example, the *lipL32* gene, culturable *Leptospira* spp., and anti-*Leptospira* antibodies have all been detected in vitreous samples obtained from eyes with chronic ERU. Despite this clear evidence of leptospiral involvement, the systemic administration of antibiotics in infected horses is ineffective at resolving ERU. This syndrome of chronic recurrent inflammation, which is unresponsive to antibiotic therapy, combined with apparent bacteria evading the immune response, is consistent with a biofilm-associated infection. The purpose of this study, therefore, was to detect the in vivo biofilm formation of *Leptospira* spp. in vitreous samples collected during vitrectomy and examined using a Warthin-Starry silver stain and immunohistochemistry. All known steps of biofilm formation were visualized in these samples, including individual *Leptospira* spp., leptospiral microcolonies and dense roundish accumulations of *Leptospira* spp. In many instances spirochetes were surrounded by an extracellular substance. Taken together, data from the present study show that ERU is a biofilm-associated intraocular leptospiral infection, which best explains the typical clinical course.

## 1. Introduction

Biofilm-associated infections are characterized by persistent and progressive disease in which the inflammatory response surrounding the biofilm plays a significant role [1,2]. Four steps of biofilm formation have been described: (1) single bacteria; (2) the formation of microcolonies; (3) a mature biofilm; and (4) the breakup of biofilm and release of planktonic bacteria [3]. In vitro biofilm formation has been described in detail for *Leptospira* spp. [4,5,6] and there is evidence of in vivo biofilm formation following experimental infections [7,8].

In horses, recurrent uveitis occurs at unpredictable intervals over a period of many years and usually leads to blindness despite intensive conservative therapy [9,10,11]. Both eyes are affected in about 25–50% of horses [12,13]. ERU affects up to 10% of all horses in Europe [14,15,16] and up to 25% in the US [17].

In European horses without a leopard coat pattern, the most effective method to prevent further episodes of uveitis and thereby preserve vision is vitrectomy [10,18,19,20,21]. After a properly performed vitrectomy, which has been routinely performed for more than 30 years in horses with ERU, the control of inflammation is seen in 90–97% of horses [10,13,21,22,23,24]. By contrast, uveitis in horses with a leopard coat pattern manifests differently. Affected horses typically do not appear to be as painful. Intraocular leptospiral infection is rarely confirmed suggesting that the etiology and pathogenesis are different from that seen in horses without a leopard coat pattern [25].

The vitreous removed during vitrectomy are intensely investigated in the hope of better determining etiology, pathogenesis and treatment strategies for ERU. Many of these studies have suggested an association between leptospiral infection and ERU [26,27,28,29,30,31]. For example, anti-*Leptospira* antibodies are regularly detected in vitreous material obtained during vitrectomies [13,15,21,32,33,34,35,36,37,38,39,40,41]. These antibodies are detected using the micro agglutination test (MAT), and various enzyme-linked immunosorbent assays (ELISA) [42,43]. In other studies, *Leptospira* spp. are cultured and anti-*Leptospira* antibodies are detected in the same vitreous samples [13,21,32,35,39,44,45,46]. In addition, *lipL32* gene or 16S-rRNA are detected by a polymerase chain reaction (PCR) in up to 70% of intraocular samples collected from affected horses [32,39,43,44,45].

Finally, scanning electron microscopy is used to reliably detect *Leptospira* spp. in vitreous material from equine eyes affected with recurrent uveitis. The leptospiral organisms were surrounded by a homogeneous granular layer, which has not been seen in *Leptospira* spp. cultured in vitro (using standard World Health Organization (WHO) strains) [47,48].

In addition to direct evidence of the leptospiral infection of vitreous samples from eyes affected with ERU, evidence of autoimmunity also exists [49]. However, Prof. Deeg’s research group [50] performed all investigations using vitreous samples, from which leptospiral infections were also regularly detected [13,21,39,41,42,43,46]. Furthermore, while vitrectomy removes the vitreous material, potential autoantigens from other tissues—especially the lens and retina—remain in the eye [18,39] which contradicts the idea of autoimmunity [39,51].

Taken together, data from numerous studies using specimens from eyes affected with ERU support the hypothesis that ERU is triggered and perpetuated by the chronic infection of the vitreous cavity with *Leptospira* spp., and that this may be eliminated by vitrectomy [13,21,32,33,36,37,39,44]. However, systemic antibiotic therapy has not been successful at controlling the inflammation seen in ERU.

The vitreous body consists of 98–99% water, contains collagen fibrils and represents a 28 mL immunological niche in horses [13,39,52,53,54]. These are optimal conditions for the biofilm production of the *Leptospira* spp. [55]. In addition, ERU exhibits all of the characteristics of a biofilm infection: chronicity, inflammation, and a high tolerance to both antibiotics [46] and the body’s immune defenses. The aim of this ex vivo study was to demonstrate, using Warthin-Starry silver stain and immunohistochemistry, the biofilm formation by *Leptospira* spp. in vitreous material obtained during vitrectomies performed on horses with ERU.

## 2. Materials and Methods

### 2.1. Positive Controls

Culture *Leptospira* spp. (WHO strains) were spread on microscope slides (Thermo Scientific Superfrost Ultra Plus; Menzel B.V. & Co.KG, Braunschweig, Germany) and allowed to dry overnight. Warthin-Starry silver stain and immunohistochemistry were then performed. The leptospiral serovars used for this study (Grippotyphosa, Bratislava, Australis, Autumnalis, Icterohaemorrhagiae and Pomona) were obtained from an accredited laboratory (State Office for Health and Food Safety, Oberschleissheim, Germany; accreditation DIN EN ISO 17025, Reg. No.: D-PL-19082-02-00).

### 2.2. Sample Selection

After careful case selection of horses whose history and clinical findings indicated *Leptospira*-induced uveitis [21], vitrectomy was performed as a therapeutic procedure at the Equine Clinic of Ludwig-Maximilians-University in Munich, Germany. All vitrectomies were performed as previously described [18]. At the beginning of surgery, about 3 mL of undiluted vitreous humor was obtained routinely from the suction line. In order to confirm intraocular leptospiral infection, an aliquot of each sample was sent to an external laboratory for MAT and PCR (Society for Innovative Veterinary Diagnostics, Seelze-Letter, German accreditation authority: D-PL-18303-02-00). Additionally, some vitreous humor was spread on microscope slides and allowed to dry overnight. Approximately 10 smears were obtained from each vitreous sample from ERU eyes. Only samples with a positive PCR result were used for the examinations (Table 1).

### 2.3. Negative Controls

For subsequent analysis using Warthin-Starry silver stain and immunohistochemistry, vitreous humor samples from clinically normal eyes in which anti-*Leptospira* antibodies could not be detected by MAT and the *lipL32* gene was not detected after 40 cycles of qPCR were used as controls (Table 1).

### 2.4. Warthin-Starry Silver Stain

A modified Warthin-Starry [56] protocol was used to identify leptospiral organisms in vitreous humor samples. The slides were first incubated in 1% silver nitrate solution (Fa. Morphisto, Offenbach a. M., Germany) for half an hour at 60 °C in the dark and then briefly rinsed in distilled water. A solution containing hydroquinone (“silver enhancer stock solution B” Fa. Morphisto, Offenbach a. M., Germany) was used to reduce the bound silver to a visible metallic form. Developer solution was prepared and preheated to 55–60 °C in a water bath and afterwards placed onto the slides for 2–3 min. The developing process was performed under visual control. The slides were briefly drained and rinsed twice for 1 min. each in warm distilled water (55–60 °C), incubated with 5% sodium thiosulfate (Fa. Morphisto, Offenbach a. M., Germany) at room temperature, and then rinsed for 3 min. under running tap water. Finally, the smears were covered with eukitt^®^ (Fa. Sigma-Aldrich Chemie GmbH, Taufkirchen, Germany) for microscopic evaluation.

### 2.5. Immunohistochemistry

The slides were pulled through the flame twice for heat fixation. All washing steps were done with phosphate buffered saline (PBS) (pH 7.4), and all incubation steps took place at room temperature. After washing once for 4 min, non-specific binding was reduced by bathing in regular protein block (Dako Protein-Block-Serum-Free, Dako GmbH, Jena, Germany) for 5 min. The slides were then incubated for one hour with a specific antibody (anti-*Leptospira* rabbit antiserum from the OIE and National Collaborating Centre for Reference and Research on Leptospirosis, Academic Medical Center, Department of Medical Microbiology, University of Amsterdam) diluted 1:5120 in Dako Diluent, Dako GmbH, Jena, Germany). After washing twice for 1 min each time, slides were incubated for 30 min. with secondary antibody (Biotinylated anti-rabbit IgG, Biozol, Eching, Germany) diluted 1:300. After washing twice more, slides were incubated with horseradish peroxidase conjugate (Streptavidin-HRP, Leika, Germany) for 20 min. After the last wash, slides were incubated for 5 min in hydrogen peroxide solution (DAB, Dako GmbH, Jena, Germany), and washed with PBS and tap water. After dehydrating in alcohol (70% alcohol, 96% alcohol, 2x isopropanol, 2x xylene) slides were covered with eukitt^®^ (Fa. Sigma-Aldrich Chemie GmbH, Taufkirchen, Germany) for microscopic evaluation. Cultured *Leptospira* spp. from an accredited leptospiral laboratory were served as positive tissue controls (State Office for Health and Food Safety, Oberschleissheim, Germany; accreditation DIN EN ISO 17025, Reg.-Nr.: D-PL-19082-02-00). Samples incubated without primary antibodies formed system controls. Vitreous samples from clinically normal eyes served as negative tissue controls (Table 1).

### 2.6. Microscopy Images Acquisition

The evaluation was performed with a light microscope (Leica DM 5000B) connected to a digital color camera (Leica DFC 450 C). Samples were routinely screened first using the 10×, then the 40×, and finally the 100× objective (with oil). Representative areas were photographed.

The results are aligned according to the steps of biofilm formation already described by Jamal et al. [3]:

1. Initial contact/attachment to the surface:
  Adhesion and cohesion; the attachment to a surface, as well as the attachment of bacteria to each other.
2. Microcolony formation:
  After stable attachment to a surface or other bacteria, the bacteria began to multiply by cell division, as well as forming extracellular polymeric substances (EPS), which led to the formation of microcolonies.
3. Maturation and architecture:
  EPS are the main components of the biofilm. Cell density in the biofilm is controlled by intercellular signaling and cell-to-cell communication (quorum sensing). This leads to the production of EPS and thus to a three-dimensional dense biofilm structure.
4. Detachment/dispersion of biofilm:
  Multiplication of bacteria in the biofilm results in further spreading and reattachment to a surface or other bacteria.

## 3. Results

Twenty-nine PCR-positive vitreous samples were selected from equine eyes clinically diagnosed with ERU (Table 1, samples 1–29) to attempt to visualize the different steps of biofilm formation. As a control, three vitreous samples were obtained from clinically healthy eyes (Table 1, samples 30–32). The vitreal leptospiral antibody titer test was positive in 28 (96.6%) of 29 samples. The negative MAT result of sample no. 21 could be due to the fact that either no antibodies had yet been formed by the time the sample was taken, or antibodies were no longer detectable, or the overall immune response was so low that it was not possible to detect antibodies by MAT.

### 3.1. Positive Controls

**Figure 1:**
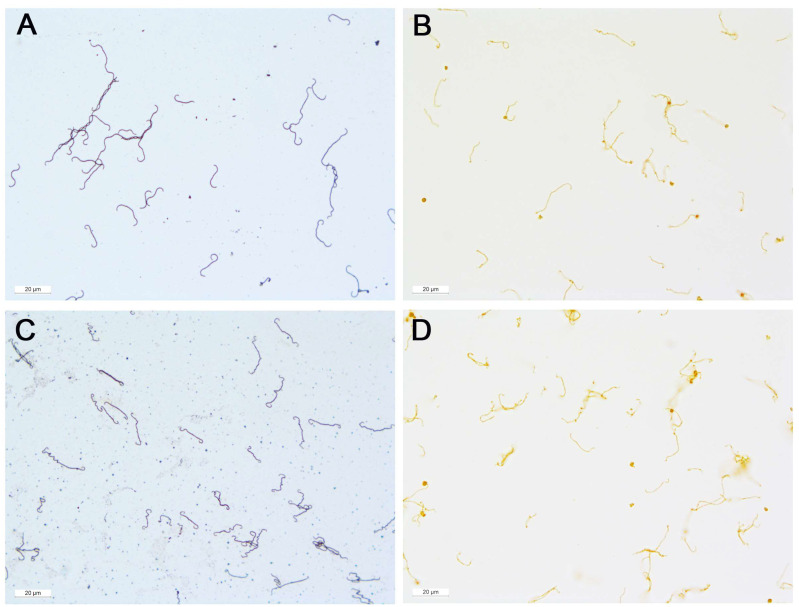
Smears from culture *Leptospira* spp. (**A**): Serovar Grippotyphosa, Warthin-Starry stain. (**B**): Serovar Grippotyphosa, immunohistochemistry. (**C**): Serovar Australis, Warthin-Starry stain. (**D**): Serovar Australis, immunohistochemistry.

The cultured *Leptospira* spp. appear as single thin corkscrew-shaped organisms in both Warthin-Starry silver stain and immunohistochemistry, as is typical for this genus. The length of the single bacteria is approximately 10 to 20 µm and they have a very thin diameter of approximately 0.5 µm. They have hooked ends on one or both sides, which distinguishes them from other spirochaetes. The individual bacteria are sometimes completely elongated, generally curved, or have more complex S, U or L shapes. Sometimes one or more bacteria lie side by side, on top of each other, or are intertwined. However, the individual bacterium is always clearly distinguishable. In some cases, the corkscrew-like coiling can be clearly seen as small bumps on the thin, filamentous leptospiral body (Figure 1). No evidence of biofilm formation was found in the cultured *Leptospira* spp.

### 3.2. Samples from ERU Eyes

*Leptospira* spp. could not be detected on every slide examined. This can be explained by the small sample volume and the inhomogeneous vitreous matrix. Nevertheless, at least one of the three steps of biofilm formation could be found in smears of each vitreous sample using both staining methods. In addition, it was possible to show all three steps of biofilm formation on the same slides. The evaluation of the slides was extremely time-consuming.

### 3.3. Steps in Biofilm Formation

**Figure 2:**
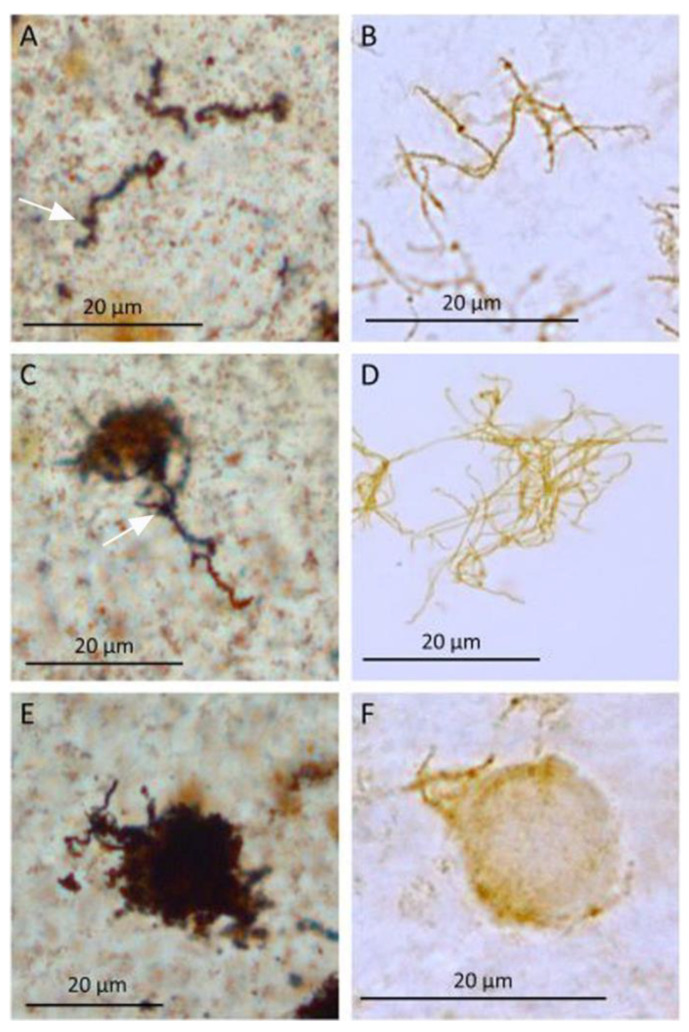
Smears from vitreous body samples from horses diagnosed with ERU. (**A**) Solitary *Leptospira* spp., Warthin-Starry stain. (**B**) Solitary *Leptospira* spp., immunohistochemistry. (**C**) Leptospiral aggregates and surrounding matrix, Warthin-Starry stain. (**D**) Leptospiral aggregates and surrounding matrix, immunohistochemistry. (**E**) Dense roundish conglomerates composed of *Leptospira* spp. and matrix, Warthin-Starry stain. (**F**) Dense roundish conglomerates composed of *Leptospira* spp. and matrix, immunohistochemistry. (Arrows: dense round structures on the *Leptospira* spp.).

1. Initial contact/attachment to the surface:

Both the Warthin-Starry silver stain (Figure 2A) and immunohistochemistry (Figure 2B) revealed solitary dark brown stained *Leptospira* spp. Individual *Leptospira* spp. showed the characteristics of spiral coils and hooked ends as described above, while two or more organisms were sometimes seen lying next to or crossing each other. Regardless, individual organisms could always be distinguished from each other easily (Figure 2A,B).

Overall, the *Leptospira* spp. from the vitreous samples of horses affected with ERU appear somewhat thicker and less delicate than the cultured *Leptospira* spp. (Figure 1). *Leptospira* spp. from vitreous samples show a significant increase in thickness (approximately 1–2 µm) especially in the Warthin-Starry silver stain, where their shape becomes more blurred. In some cases, *Leptospira* spp. are covered by granular structures. In addition, a stronger staining of the leptospiral environment can be seen, which is represented by the light brown (IHC) or dark brown (Warthin-Starry silver stain) stained and partially granulated matrix (Figure 2).

2. Microcolony formation:

Individual, well-defined *Leptospira* spp. and dense variably sized aggregates of *Leptospira* spp. were seen using either silver stain or the antibody against Grippotyphosa (Figure 2C,D). The aggregates were round, oval or polygonal with some having “frayed” borders consisting of individual intact or fragmental *Leptospira* spp. (Figure 2C,D). A variably intensely positive, immunohistochemical, reacting homogeneous matrix was evident between the densely packed *Leptospira* aggregates (Figure 2D). Similarly, in the Warthin-Starry silver stain, a slightly weaker stained inhomogeneous granular matrix appeared between the denser bacteria, which was strongly stained (Figure 2C).

3. Maturation and architecture:

The leptospiral aggregates and the secreted substance condensed such that individual *Leptospira* spp. could not be distinguished from each other (Figure 2E,F). These condensed structures were approximately round with a diameter of approximately 5 to 20 µm and were clearly distinguishable from the surroundings. Occasionally, individual *Leptospira* spp. protruding from the aggregates could be identified by their characteristic corkscrew-like coiling and/or hooked ends (Figure 2E,F).

### 3.4. Negative Controls

Vitreous samples from clinically healthy equine eyes were negative for both leptospiral PCR and leptospiral antibody titers (MAT) (Table 1, sample 30–32). Neither *Leptospira* spp. nor evidence of biofilm formation were detected in these samples using Warthin-Starry silver stain or immunohistochemistry.

## 4. Discussion

Descriptions of in vitro biofilm formation have been published for *Borrelia* spp. [57] and *Leptospira* spp. [5,6]. In vivo biofilm formation is described in humans with lymphoma [58] or Lyme borreliosis [59]. Although the in vivo biofilm formation by *Leptospira* spp. has been suspected in renal tubules [5,60], to date it is found exclusively after experimental infections [7,8]. The attachment of bacteria to surfaces is considered to be of great importance for biofilm formation. However, pathogens can also attach to each other and can form a biofilm without being attached to another surface or host tissue. A biofilm can occur in liquid or mucous media [2,3,61,62,63]. The mechanical or surgical removal of the biofilm is described as the best way to eliminate chronic biofilm-associated infections [64,65,66].

To the authors’ knowledge, the present study was the first to detect *Leptospira* spp. and different steps of biofilm formation in vitreous samples of horses with ERU using immunohistochemical analysis and Warthin-Starry silver staining. Previously, IHC analyses were performed exclusively on fixed tissue sections. In addition, MAT, ELISA, fluorescent antibody tests, dark-field microscopy, culture, PCR, and histopathology with special stains are all used to identify anti-*Leptospira* antibodies, *Leptospira* spp., or leptospiral DNA in the tissues or body fluids of dogs [67,68,69]. Whereas, in hamsters, the leptospiral injury of the kidney has been investigated using culture, histology, MAT, serum creatinine concentrations, and immunofluorescent staining [70]. In the present study, tissue sections are not available. Rather, the undiluted vitreous samples of 2–3 mL per patient were utilized. This necessitated the development of a method that does not notably alter spirochete morphology but in which the organisms are reliably stained. Two different techniques are used for this purpose:

Silver plating [71,72,73] is considered a reliable technique for the demonstration of spirochetes. Difficulties with this method arise, however, when only a few organisms are present, since leptospiral fragments cannot be detected. Therefore, in the present study, we used a rabbit anti-*Leptospira* antibody and a modified immunohistochemical protocol as a supplemental method for the morphological visualization of *Leptospira* spp. and of the various steps of biofilm formation. IHC has been previously described as a specific detection method of leptospiral antigens in various tissue samples and species and for studying biofilm formation in vivo of pathogenic *Leptospira* spp. [74,75,76].

To the authors’ knowledge, this is also the first morphological description of the steps of biofilm formation of *Leptospira* spp. in vivo; however, the same steps of biofilm formation described for bacteria in general [3,64,77] were demonstrated here using Warthin-Starry silver stain and IHC. In addition, the morphological characteristics of in vivo biofilm formation demonstrated in the present study were similar to those shown in vitro for saprophytic and pathogenic *Leptospira* spp., using transmission electron microscopic studies [5]. Individual *Leptospira* spp., cell aggregates, microcolonies, and, ultimately, mature biofilm structures were all visualized in the present study. In accordance with Ristow et al. [5], bacterial colonies in the mature biofilm were densely packed, surrounded by an amorphous mass, and formed an approximately round structure.

In vitro*, Leptospira* spp. are capable of biofilm formation within a few days [5,6] with the first stage requiring cohesion (binding of bacteria to each other) and adhesion (binding of bacteria to a surface) [78,79]. After individual bacteria assemble into microcolonies they surround themselves with an amorphous polymer matrix [62], with the mature biofilm structure taking on a round shape and individual *Leptospira* spp. no longer recognizable [6]. Because the polymer matrix of the biofilm is produced by the bacteria themselves it is detectable using antibodies directed against *Leptospira* spp. In the present study, bacterial cohesion was morphologically evident by silver staining and IHC labeling; however, other structures of the vitreous (collagen fibrils, cells, and inflammatory products formed as a result of uveitis) were not stained. IHC was previously described as a specific detection method of leptospiral antigens in various tissue samples and species and for studying biofilm formation in vivo of pathogenic *Leptospira* spp. [74,75,76].

Canine renal tissues were stained with specific leptospiral antigens and were further investigated with the silver staining method. Therefore, the results of Wild et al. [80] show that immunohistochemistry is a helpful method in the diagnosis of canine leptospirosis. Additionally, immunohistochemistry and the Warthin-Starry stain showed that pathogenic *Leptospira* spp. were present on the surface of pulmonary epithelium [81].

The slides on which no *Leptospira* spp. were found can be explained, among other things, by the fact that the vitreous is very inhomogeneous, consists of 98% water, and only about 1 mL of the vitreous material is available undiluted for smears. All other studies detecting *Leptospira* spp. use tissue guided by other structures and serial sections are available [76,81].

Six criteria have been established to describe biofilm-associated infections: four original criteria by Parsek and Singh [82], and two additional criteria by Hall-Stoodley and Stoodley [83]. It is worthwhile to consider each of these criteria individually as they relate to observations in the present study.

1. Infecting bacteria are bound to a substrate or a surface [82]:

Although attachment of *Leptospira* spp. to a surface is not demonstrated in the present study, this is not expected since the vitreous body is composed largely of water. In addition, biofilm formation without attachment to a surface has been described. It is likely in these situations that this occurs via the cohesion of bacteria to each other. For example, a *Pseudomonas aeruginosa* biofilm without surface attachment is found in tracheobronchial secretions [2,3,61,62]. It is also possible for bacterial adhesion to occur at a microscopic (cellular or subcellular) rather than tissue level, for example, in the vitreous cavity to collagen fibrils [53,54] present in the normal and inflamed vitreous, or to a serum amyloid A [84,85] present in inflamed vitreous. In aquatic environments, plant fibers represent comparable surfaces on which *Leptospira* spp. biofilm formation can begin [86]. Regardless of whether or not fibrils initially serve as surfaces for the attachment in the vitreous cavity, leptospiral biofilm formation may additionally occur freely in aqueous vitreous phases.

2. The direct examination of infected tissue reveals bacteria living in cell aggregates or microcolonies surrounded by extracellular matrix [82]:

Immunohistochemical examination of vitreous samples from eyes with ERU in the present study reveals various steps of biofilm formation, including cell aggregates and microcolonies. In each case, bacteria are surrounded by an extracellular matrix that is also immunopositive. This extracellular matrix surrounding *Leptospira* spp. is also found ultrastructurally in vitreous samples from equine eyes affected with ERU [47,48].

3. Biofilm-associated infections are generally confined to a specific location (although bacterial dissemination may occur, it is considered a secondary phenomenon) [82]:

Leptospiral infection in horses with ERU is an intraocular infection exclusively detectable in the aqueous humor and vitreous samples [13,39]. Since both leptospiral culture and PCR are positive more frequently with vitreous material than with the aqueous humor, there is much to suggest that *Leptospira* spp. persist in the vitreous cavity [13,39]. The detection of *Leptospira* spp. or its DNA within the aqueous humor may represent organisms that have entered the anterior chamber of the eye from the vitreous humor between the zonular fibers of the lens since there is limited exchange of water between the two humors. By contrast, the persistence of *Leptospira* spp. in the lens or the highly vascular uvea seems unlikely. Similarly, there is no evidence for the dissemination of *Leptospira* spp. from the eye to other systemic sites. Further supporting the hypothesis that leptospiral infection is limited to the vitreous is the knowledge that the vitrectomy reliably eliminates ERU [13,18,21,39]. After vitrectomies, intraocular antibody titers decrease continuously and after one year MAT becomes negative, and *Leptospira* spp. cannot be cultured and neither the *lipL32* gene nor 16S rRNA can be detected by PCR in intraocular samples [39].

4. Biofilm-associated infections are impossible or difficult to eliminate using antibiotics, to which the responsible organisms are sensitive when in their planktonic or free-living state [82]:

The systemic administration of antibiotics effective against *Leptospira* spp. are ineffective at controlling ERU. Despite vitreal enrofloxacin concentrations above the minimum inhibitory concentrations (MIC) for *Leptospira* spp., these organisms could be cultured from vitreous material [46]. In fact, cultures were only slightly less frequently positive in enrofloxacin-treated (30%) than in untreated (54%) horses. *Leptospira* spp. have also been cultured in some cases from the irrigation fluid collected during vitrectomies, in which the removed vitreous material is diluted approximately 10-fold and exposed to 0.8 mg/mL of gentamicin [40,87]—approximately 100-fold higher than the MIC [88].

5. No organism can be cultured despite a strong presumption of infection with the pathogen of interest [83]:

Conditions for the culture of *Leptospira* spp. were demanding. Even with the optimal collection techniques of undiluted vitreous samples and the immediate sterile inoculation into a transport medium, *Leptospira* spp. were cultured from only 53% of the samples [13,39]. This percentage is comparatively high. However, the positive culture result was often obtained only after several months. In other studies, *Leptospira* spp. could be cultured in only a small percentage of the vitreous samples examined [32,35,44]. Many other investigators failed to culture *Leptospira* spp. [26,31,89,90], which led to the hypothesis that ERU is an autoimmune disease [90,91,92].

6. Ineffective immune response as evidenced by bacterial aggregates surrounded by inflammatory cells within host tissue [83]:

The vitreous body is 98%–99% water, but also contains collagen fibrils, occasional cells, and hyaluronic acid [52,53,54]. As such, vitreous material is not a typical “tissue” but rather a somewhat heterogeneous viscous liquid. The histological preparations of vitreous specimens often do not show stainable *Leptospira* spp. Therefore, an inflammatory reaction surrounding the bacterial aggregates is not present in the same form seen in other tissues. However, when considering the closest vascularized tissue—the uvea, an increase in inflammatory cells is noted, especially in the ciliary body [93,94], but also within the vitreous cavity [47,48,53,95]. Despite high antibody titers and the presence of macrophages within the vitreous, vitreal *Leptospira* spp. are not eliminated. On the contrary, high vitreal anti-*Leptospira* antibody titers increase the probability of successful leptospiral culture from that sample [13,39,47]. If, as suspected, the dense round structures seen in the images by Brandes et al. [47] are biofilm, it seems possible that these structures may not be completely eliminated by macrophages.

Taking into account that the vitreous material as a heterogeneous viscous liquid cannot be equated with other tissues in every point, ERU fulfills all criteria described for biofilm-associated infections.

Neutrophil extracellular traps (NETs) have also been detected in vitreous samples from eyes affected by ERU [96]. Bacterial infections can initiate the formation of NETs, particularly when phagocytosis fails to eliminate the pathogen. Biofilms appear to be a particularly notable trigger for NET release. NETs, in turn, can stimulate biofilm formation [97,98]. Thus, a mutual positive feedback mechanism exists here, and mature, stable biofilms may be surrounded by larger amounts of NETs [98]. Although in the present study no NETs were visualized, this warrants further assessment in future studies.

Boundary tissues or “locus minoris resistentiae” are often located within avascular tissues of eye, kidney, joint, heart valves, arteries, and skin where there are reduced or absent inflammatory responses. As a result, pathogens can colonize and often persist at these sites [99]. It is interesting to consider the vitreous as a potential locus minoris resistentiae, with the capillaries of the ciliary body acting as the most likely site of entry into the vitreous cavity for *Leptospira* spp. As such, the vitreous body may be thought of as representing a 28 mL avascular immunological niche [13,39].

## 5. Conclusions

The clinical signs and chronic disease course of ERU, as well culturable *Leptospira* spp. in vitreous samples from these eyes, despite high vitreal antibody titers, apparent immune evasion, and ineffectiveness of antibiotics, fulfill all the criteria of a biofilm-associated infection. The previously known steps of biofilm formation have hereby been demonstrated in vitreous samples from equine eyes suffering from recurrent uveitis using both Warthin-Starry silver stain and immunohistochemistry. Thus, we conclude that ERU is a spontaneous disease due to in vivo biofilm formation by *Leptospira* spp. Future studies should further differentiate biofilm formation in the equine vitreous, analyze the composition of this biofilm, and provide insights for other biofilm-associated infections.

## Figures and Tables

**Table 1:** Signalment of equine patients, clinical diagnosis and laboratory findings of vitreous samples.

Sample	Signalment	Clinical Diagnosis	*lipL32* Gene PCR Result (Ct Value)	Vitreal Leptospiral Antibody Titer (MAT) ^1^
1	4-year-old warmblood gelding	ERU	Positive (Ct 37)	*Grippotyphosa* 1:3200
2	8-year-old Icelandic horse mare	ERU	Positive (Ct 40)	*Grippotyphosa* 1:400
3	7-year-old Friesian mare	ERU	Positive (Ct 29)	*Grippotyphosa* 1:3200
4	7-year-old warmblood mare	ERU	Positive (Ct 37)	*Grippotyphosa* 1:800
5	15-year-old warmblood gelding	ERU	Positive (Ct 38)	*Grippotyphosa* 1:400
6	10-year-old Friesian gelding	ERU	Positive (Ct 30)	*Altoduro* 1:400
7	6-year-old warmblood mare	ERU	Positive (Ct 32)	*Grippotyphosa* 1:3200
8	7-year-old warmblood mare	ERU	Positive (Ct 35)	*Grippotyphosa* 1:3200
9	4-year-old thoroughbred gelding	ERU	Positive (Ct 33)	*Grippotyphosa* 1:3200
10	6-year-old warmblood mare	ERU	Positive (Ct 34)	*Grippotyphosa* 1:3200
11	5-year-old Icelandic horse gelding	ERU	Positive (Ct 33)	*Altoduro* 1:100
12	5-year-old warmblood stallion	ERU	Positive (Ct 33)	*Grippotyphosa* 1:3200
13	5-year-old warmblood gelding	ERU	Positive (Ct 38)	*Grippotyphosa* 1:3200
14	10-year-old warmblood gelding	ERU	Positive (Ct 39)	*Grippotyphosa* 1:800
15	8-year-old warmblood mare	ERU	Positive (Ct 38)	*Grippotyphosa* 1:800
16	5-year-old warmblood mare	ERU	Positive (Ct 35)	*Grippotyphosa* 1:3200
17	8-year-old warmblood gelding	ERU	Positive (Ct 35)	*Grippotyphosa* 1:200
18	5-year-old warmblood gelding	ERU	Positive (Ct 32)	*Grippotyphosa* 1:200
19	5-year-old warmblood mare	ERU	Positive (Ct 33)	*Grippotyphosa* 1:1600
20	4-year-old warmblood gelding	ERU	Positive (Ct 33)	*Grippotyphosa* 1:100
21	3-year-old warmblood mare	ERU	Positive (Ct 38)	Negative
22	10-year-old warmblood gelding	ERU	Positive (Ct 36)	*Grippotyphosa* 1:3200
23	6-year-old warmblood mare	ERU	Positive (Ct 36)	*Grippotyphosa* 1:3200
24	7-year-old purebred Spanish stallion	ERU	Positive (Ct 39)	*Grippotyphosa* 1:3200
25	10-year-old purebred Spanish mare	ERU	Positive (Ct 39)	*Australis* 1:3200
26	15-year-old warmblood mare	ERU	Positive (Ct 39)	*Grippotyphosa* 1:3200
27	8-year-old Welsh pony mare	ERU	Positive (Ct 32)	*Grippotyphosa* 1:3200
28	7-year-old warmblood gelding	ERU	Positive (Ct 29)	*Pomona* 1:3200
29	12-year-old warmblood gelding	ERU	Positive (Ct 35)	*Grippotyphosa* 1:3200
30	8-year-old warmblood gelding	Normal ^2^	Negative	Negative
31	23-year-old Haflinger mare	Normal ^2^	Negative	Negative
32	17-year-old warmblood gelding	Normal ^2^	Negative	Negative

^1^ Only the serovar with the highest titer is shown. ^2^ Clinical examinations revealed no signs of ERU.
